# Integration of Break Dance and Computational Physics: An Interdisciplinary Approach to Contextualized Teaching

**DOI:** 10.12688/f1000research.166881.1

**Published:** 2025-10-17

**Authors:** Juan José Velandia Huérfano, Diego Fernando Becerra Rodriguez, Óscar Rafael Boude Figueredo, Ana Dolores Vargas Sánchez, Mario Humberto Ramírez Díaz

**Affiliations:** 1 Facultad de Educación, Universidad de La Sabana, Chia, Cundinamarca, 250001, Colombia; 2 Centro de Investigación en Ciencia Aplicada y Tecnología Avanzada, Instituto Politecnico Nacional, Mexico City, Mexico City, 11500, Mexico

**Keywords:** Physics Education, Educational Technology, Contextualized Learning, Non-formal Education, Dance.

## Abstract

**Background:**

This study presents an interdisciplinary pedagogical approach aimed at contextualizing the teaching of classical mechanics through the computational analysis of Break Dance movements. Situated within a constructivist framework, the research explores how culturally embedded practices—specifically urban dance—can serve as a medium for fostering conceptual understanding of physics in non-formal educational settings. By leveraging the artistic and kinesthetic dimensions of Break Dance, the study seeks to bridge the gap between abstract scientific content and learners’ lived experiences.

**Method:**

A mixed-method, exploratory design was employed with a purposive sample of ten dancers (aged 13–30) affiliated with a community-based urban dance school in Bogotá, Colombia. Over the course of six three-hour sessions, participants engaged in movement analysis using Tracker video software, supported by pre- and post-intervention semi-structured interviews. The research design incorporated thematic analysis to interpret qualitative data, complemented by the kinematic study of body movement parameters such as angular velocity and center of mass.

**Results:**

Findings indicate a marked cognitive shift among participants from intuitive and superficial conceptions to a more technical and applied understanding of physics principles. The integration of computational tools allowed dancers to visualize and internalize biomechanical variables relevant to their performance. Participants reported enhanced bodily control, injury prevention, and aesthetic execution, alongside increased motivation and collaborative learning.

**Conclusions:**

The study concludes that embedding scientific content within culturally relevant, embodied practices—mediated by educational technologies—can significantly enhance learning outcomes in physics. The use of Tracker software not only demystified abstract concepts but also redefined physics as accessible and contextually meaningful. These results underscore the pedagogical potential of transdisciplinary, arts-integrated methodologies to foster inclusive, situated, and cognitively rich science education in non-traditional environments.

## Introduction

Break Dance is a dance style that originated in the Bronx, New York City, in the 1970s and is regarded as an important cultural movement globally, notably in Latin America. This style is acknowledged for its challenging acrobatic and balancing maneuvers, necessitating exceptional bodily control and precision. Furthermore, Break Dance serves as a non-violent competitive outlet, facilitating the channeling of energy, expression of discontent, and fostering a feeling of solidarity among youth, particularly in the face of socioeconomic adversity. ^1^

Since its inception, breaking has transformed into a renowned amalgamation of art and sport, garnering global repercussions and a following while maintaining its intrinsic core and mode of expression. This expansion results from events like as the “Red Bull BC One,” ^2^ which has internationally showcased Break Dance, along with its representation in films and television programs that enhance its visibility. Nonetheless, its significance in sports and the arts has elevated it to the status of an Olympic sport for the Paris 2024 Games. ^3^ This dancing form incorporates intricate motions such as spins, force interactions, angular momentum, and displacements, making it a subject of physical study. Furthermore, these movements can be examined and assessed utilizing technology computational methods that may enhance teaching and learning processes. ^4^

On the other hand, considering that the constructivist learning model seeks to encourage active student participation, promoting meaningful learning that leads to a deeper understanding, particularly in the field of physics, the Tracker software enables the analysis of human body movement through its measurement and evaluation functions, making it a valuable tool for teaching physics. ^5, 6^

This research seeks to integrate Break Dance, computational physics, and physics education through an interdisciplinary framework, considering the diverse contributions of dance to scientific endeavors and its pivotal role in physics instruction, while emphasizing the development of activities that transcend rote memorization of formulas and mechanical exercise execution. ^7^ In this context, computational analysis of the Tracker software is employed, leveraging the distinctive movements of this dancing form as an educational resource for imparting key notions of classical mechanics. Consequently, the research question emerges: How can computational physical analysis utilizing Tracker software examine specific Break Dance actions to enhance dance skills through the application of classical mechanics?

This research aims to enhance understanding of Break Dance physics while offering an unconventional pedagogical approach within traditional physics education, emphasizing the discipline, culture, and aesthetic of human movement. Furthermore, according to the concept posited by ^8^ regarding dance as a medium for the investigation of physical principles, it is essential to comprehend this domain through human movement while integrating the inherent social and cultural influences stemming from each dancer’s experiences and acquired knowledge.

## Methodology

This study employed an exploratory mixed-methods design based on the pragmatic paradigm, which facilitated the combination of qualitative and quantitative data to understand the multifaceted nature of art instruction in community dance settings. The study used a purposive sample of ten dancers (eight men and two women), aged between 13 and 30, who had been associated with the institution for periods ranging from four months to nine years. Regarding their educational level, three participants completed primary basic education, four hold professional technical degrees, and three are pursuing undergraduate studies. In addition to these, four have formal academic training in arts and/or sports, which added an important dimension of professionalization to the group. All participants provided written informed consent prior to their inclusion in the study. Additionally, given that one of the participants was a minor, written informed parental consent was obtained to ensure full ethical compliance with research standards.

The study was conducted with the “Soul Strong Crew” group, a community urban dance collective located in District 18 of Rafael Uribe Uribe in Bogotá D.C., Colombia. Since its founding in 2014, the group has actively participated in art education, competitions, and community outreach activities, positioning dance as a vehicle for social transformation. Their pedagogical practices emphasize the power of experiential learning and community dialogue, based on the conviction that young people are agents of change, and that artistic talent is a form of resistance and resilience in marginalized contexts.

### Type of study

This study employed an exploratory mixed-methods approach, prioritizing qualitative analysis while using quantitative metrics, to examine the multidisciplinary fusion of breakdancing, computational physics, and physics education. The study sought to investigate how the computational analysis of distinctive Break Dance movements, utilizing the open-source program Tracker, may function as an educational tool for fundamental principles of classical mechanics while concurrently enhancing dance technique. This method facilitated the methodical expression of embodied movement and scientific understanding. The mixed methods strategy facilitated the triangulation of physical, educational, and performative data, consistent with ^9^ framework for addressing complex educational issues through methodological complementarity.

### Ethical considerations

This research examined moral and legal rules concerning the ethical treatment and care of participants in the studies. ^10^ This study received approval from ACT 22-2022 (June 13, 2022) by the Research and Ethics Subcommittee of the Faculty of Education at the University of La Sabana. The document indicates that *“The Research and Ethics Subcommittee of the Faculty of Education has approved the project EDULCN-1-2022; Collaborative Learning, Computational Physics, and Experimental Setup Construction in the Approach to the Study of Renewable Energies, directed by Professor Diego Fernando Becerra (corresponding author). The suggestion is regarded favorably due to its significance. Under the guidance of project leader Professor Diego F. Becerra, his research focus within the Technologies for the Academy (Proventus) group, and his involvement in the Bachelor of Science in Natural Sciences and the Master’s program in Educational Innovation mediated by ICT”.* This study adheres to the global research conduct standards outlined in the UNESCO Universal Declaration on Bioethics and Human Rights, as well as the ethical principles set forth by the Ethics Committee of the University of La Sabana. The research emphasizes the importance of telling participants about the study, detailing their involvement, safeguarding their identity, and assuring that no bodily or emotional harm would ensue.

Moreover, it is stated that the research is undertaken for the advancement of the academic community, that the data is safeguarded and utilized exclusively for educational research purposes, and that a conscientious approach is maintained, remaining receptive to inquiries from participants and community members. Moreover, only data from minors whose parents have properly signed the informed permission form will be used into the study.

### Method

The research was conducted in phases over six sessions, each lasting three hours, during the dance training organized by the institution. The academy’s instructors conduct these dancing sessions, allowing for observable incremental advancement in each student’s artistic and athletic training. In these environments, aspects such as stage presence, precise execution of motions, the significance of physical activity, reflections on sports and competition, and opportunities for interaction and the exchange of experiences and knowledge are addressed. The sessions were executed in the following manner:

**Session 1:** The initial session offered a comprehensive contextualization of the research objective, outlined key concepts to be explored, detailed the planned activities for each session, and established the criteria for result categorization, underscoring that participant confidentiality was paramount. Furthermore, it was indicated that the study’s purpose was associated with the enhancement of the classes they participate in, without disrupting any scheduled school activities. A context recognition was performed, wherein each participant was inquired about their age, duration of attendance at the school, academic history, preferences related to dancing, and their personal and professional training.

**Session 2:** The second session involved conducting a preliminary investigation focused on early perceptions regarding the physics of motion. A question ladder (refer to Table 1), validated by expert researchers from the Faculty of Education at the University of La Sabana, was employed as an instrument, concentrating on the physics of motion, center of mass, circular motion, and the perceived correlation between physics and the enhancement of their technique. Data collection was conducted via audio recordings of the verbal responses given by each participant.

**Table 1.** Hierarchy of inquiries for elucidating preliminary viewpoints.

Phase	Inquiry
Identification	What do you understand by the physics of motion?
Identification	What do you understand by center of mass and circular motion, additionally how do you think it applies to Break Dance movements?
Exploration	Imagine you are a Break Dance coach with knowledge of physics. How do you think you could use that knowledge to improve your movements?
Reflection	How do you think physics can influence your Break Dance movements?

Subsequently, when the initial perspectives of all participants were collected, an introduction to the Tracker software was conducted, explaining the analysis that can be performed on a video and how the study of participants’ movements would be carried out through recordings.

**Session 3:** Some recordings were made of the participants performing specific Break Dance moves. First, emphasis was placed on some circular movements (“windmills” or “headspins”) and frozen positions (“Freezes”), with the recordings being made from multiple angles in order to have complete coverage of the movement. Videos were made of both dancers with years of experience in Breaking and students who had been in their training process for a short time, in order to compare positions and, through the study of those movements, help the participants improve their dance steps.

**Figure 1.**
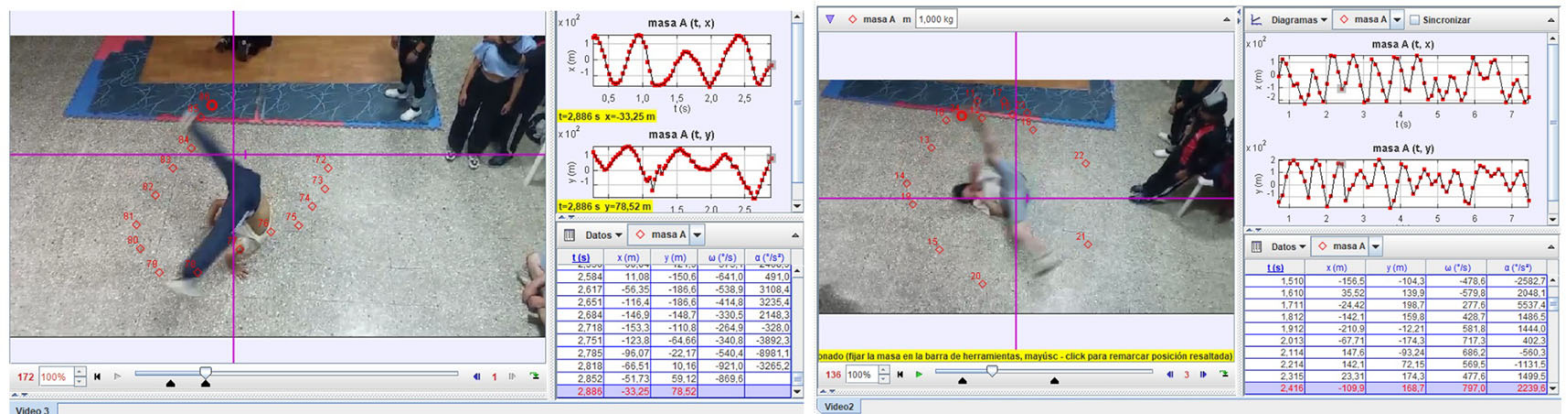
Simulation example in Tracker software.

**Figure 2.**
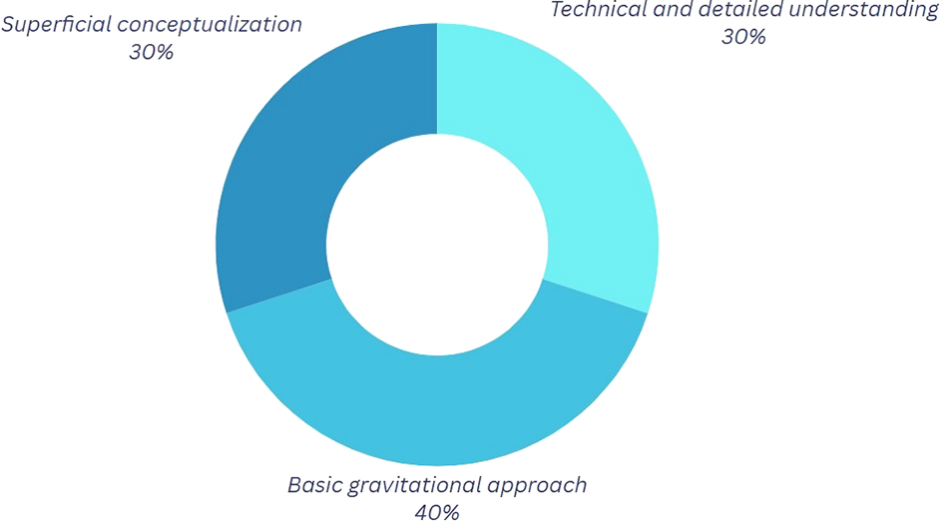
Graph corresponding to the answers to question 1, initial phase.

**Session 4 and 5:** As can be seen in Figures 1 and 2, the analysis of these videos was carried out using the Tracker software. The analysis included a measurement of kinematic parameters such as angular velocity, centripetal acceleration, and the trajectory of the center of mass for circular movements. However, for the frozen positions, the location of the center of mass was determined and emphasis was placed on the stability of those positions. In that sense, it can be evidenced that the Tracker software consolidates itself as a technological tool that contributes to the modernization of knowledge ^11^ were, with the help of innovative technologies, physics can be taught in unconventional environments, different from a school or a university, with prior study of the context.

Following the analysis, two feedback sessions were conducted with the participants of the artistic group to discuss the findings and elucidate the application of these physical ideas to the specific movements demonstrated in the videos. The lessons served as instructional interventions, enabling the dancers to acquire a profound comprehension of the physics behind their motions and its potential to enhance their performance. A knowledge-sharing session was conducted in which the dance school instructors correlated concepts and aspects of physics with their movement execution to elucidate the information for all attendees.

**Session 6:** Subsequently, a second series of questions was administered to elucidate the ultimate viewpoints within the responses. This questionnaire had inquiries that examined the shift in perspective regarding the physics of movement, the application of these new insights in practice, and the perception of the relationship between the analyzed physical variables and dance performance.

The qualitative data from the question ladders were examined using the “Thematic Analysis” method, which, according to, ^12^ seeks to find patterns in the development of dancers’ cognition. Similarly, the qualitative data were classified according to the discovery of emergent patterns, facilitating a systematic and foundational categorization in the analytical process. Furthermore, all previously described methodological stages were executed in strict adherence to the ethical principles established by “Soul Strong Crew,” with particular emphasis on data confidentiality and participant welfare.

The data from reseaerch, Integrating Breakdance and Computational Physics: An interdisciplinary approach to contextualized teaching, have been published by, ^13^ and are located in the availability section.

## Results

### Phase 1. Preliminary assessments

Concerning the outcomes, it is essential to note that they are categorized into two components. The first component analyzes the dancers’ initial notions to assess their knowledge of the concepts, based on the leading questions in Table 1. During the performance of the dance, physical concepts are involved that, although often understood theoretically, are not often consciously acknowledged while performing the dance steps.

The responses are categorized by question, considering the idea and practical application of physics in this dancing form. The data were systematically organized as follows:

*Question 1:* What do you understand by the physics of motion?

Analysis of the graphs pertaining to the first component (refer to Figure 2 and Table 3) reveals that 60% of participants acknowledge a certain significance of physics in Break Dance; however, a notable disparity exists in the level of comprehension regarding the application of these principles. Participants employing a more technical methodology often integrate variables such as forces and postures into their discussions to enhance movement execution and mitigate injury risk. This aspect is particularly pertinent, as proper posture and accurate execution of movements can aid in the prevention of injuries that, if sustained, could hinder artistic-sporting development, obstruct participation in competitions for an extended duration, or even profoundly alter one’s lifestyle.

**Table 2.** Ladder of questions to make the final perspectives and knowledge visible.

Phase	Inquiry
Identification	How has your understanding of the physics of motion changed?
Identification	How have the results of the analysis changed the way you perform certain movements?
Exploration	How are the analyzed physical variables related to your improvements in Break Dance?
Reflection	How do you think this knowledge will influence your practice and execution of Break Dance?

**Table 3.** Classification of the outcomes for question 1.

Category	Explanation
Technical and Detailed Understanding In this category	Participants mention elements such as force interaction, speed, rotations, and physical variables.
Basic Gravitational Approach In this category	Participants tend to give answers that involve the acceleration of gravity; however, the explanation provided is limited regarding its influence on motion.
Superficial Conceptualization	In this category, explanations that do not mention technical details are highlighted.

*Question 2:* What do you understand by center of mass and circular motion, and how do you think it applies to Break Dance movements?

**Figure 3.**
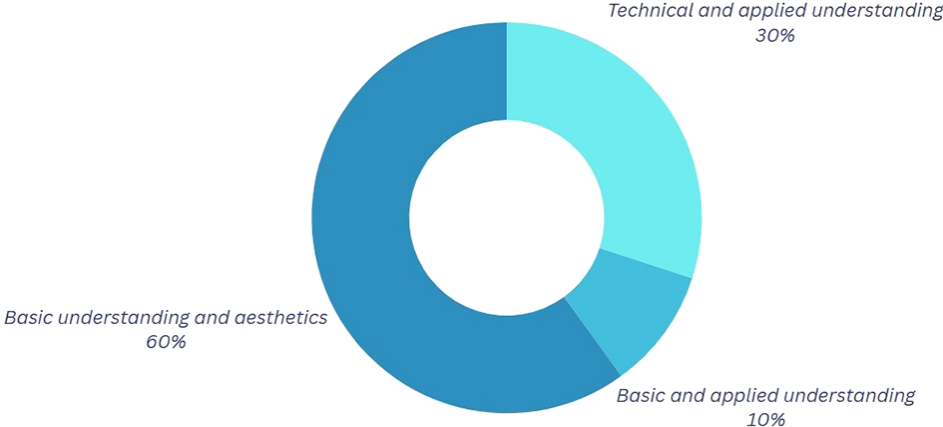
Graph corresponding to the answers to question 2, initial phase.

Participants who emphasize the visual or aesthetic aspects of their movements, as seen in Figure 3, connect physics to the favorable perception of the audience observing them during competition or training. At this juncture, certain dancers articulate the quintessence of their performance, wherein personal character, emotions, style, and technical proficiency coalesce. These aspects collectively enable each dancer to cultivate a distinctive interpretation of the movements, producing a visual effect on both the judges and the competitors. This implies that, within this framework, the physics of movement can be examined not solely through a scientific lens but also through its expression in more harmonic and beautiful forms, rendering it more tangible and accessible.

*Question 3:* “Imagine you are a Break Dance coach with knowledge of physics.” How do you think you could use that knowledge to improve your movements?

**Figure 4.**
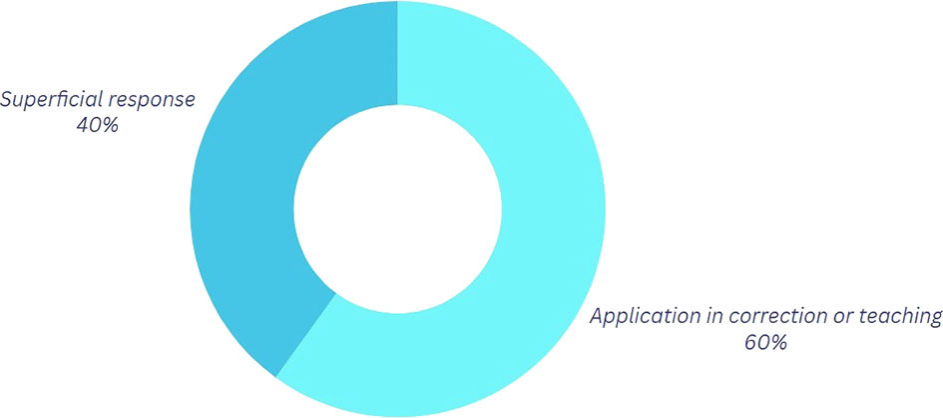
Graph corresponding to the answers to question 3, initial phase.

Concerning this inquiry, as illustrated in Figure 4 and based on the classification presented in Table 5, 60% of the participants assert that employing physics to refine their movements is a feasible approach when delivering feedback on their choreographies or specific steps. The pedagogical component is readily observable, as teaching and correcting others through physics facilitates community growth and fosters a sense of belonging within a dance “crew,” thereby integrating the social and emotional dimensions intrinsic to the culture into the learning process.

**Table 4.** Classification of the outcomes for question 2.

Category	Explanation
Technical and Detailed Understanding In this category	In this category, participants mention in detail and technically some definitions of the mentioned physical variables.
Basic Gravitational Approach In this category	In this category, the participants have a basic conceptual idea; however, they mention important elements in the application of these when performing their dance.
Superficial Conceptualization	In this category, participants have a basic conceptual idea; however, the aesthetics of the movements stand out without delving into the physical application.

**Table 5.** Categorization of the outcomes for question 2.

Category	Explanation
Application in correction or teaching	In this category, physics is used to correct movements and proper teaching to others is mentioned based on what has been learned in physics.
Superficial response	They are responses without a clear or specific focus.

*Question 4:* How do you think physics can influence your Break Dance movements?

**Figure 5.**
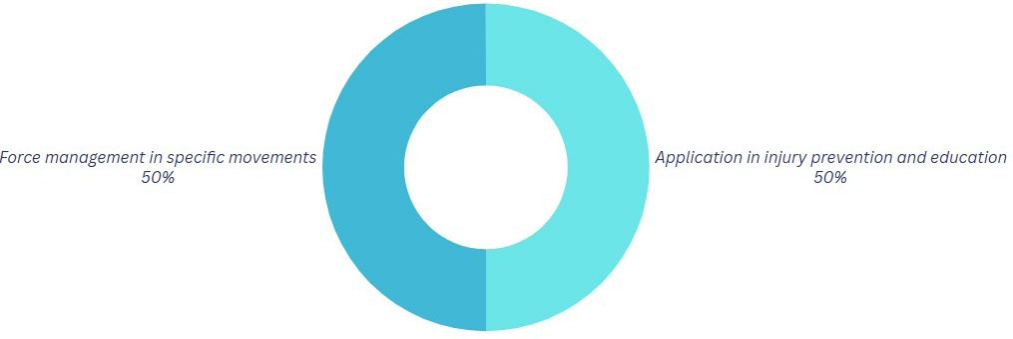
Graph corresponding to the answers to question 4, initial phase.

Figure 5 illustrates that injury prevention is a crucial part, as physics can play a role in mitigating injuries, while also acknowledging physics as a factor inherent in every activity. ^14^ asserts that scientific research on dance yields significant enhancements in technique, health, and overall well-being of the dancer. From that viewpoint, an injury would result in a postponement of the breaker’s artistic-sporting progression, along with significant emotional and psychological repercussions. Consequently, any knowledge or instrument that reduces the likelihood of damage for participants is of paramount significance. Conversely, the analysis of the responses indicates that the terms “acceleration due to gravity” and “forces” are frequently referenced, implying that participants commonly incorporate this terminology in their training development.

The practical application of physics may differ according on the degree of technical comprehension. This suggests that enhanced training in physics could not only refine movement execution but also provide instructors with superior pedagogical skills, enabling them to teach this dance style more effectively. The study primarily focuses on imparting concepts via computational analysis utilizing Tracker software. In this context, the two feedback and instructional sessions about the mechanics of their movements yielded significant information that bolsters the research. Following socialization and instruction, it became feasible to integrate physics education within the context of dance, which proved to be motivating for the participants, as they perceived that learning physics necessitated a classroom environment and the manipulation of intricate formulas.

### Phase 2. Final perceptions

In phase 2, the suggested question ladder was employed to elucidate the dancers’ knowledge and perceptions post-instruction (see to Table 2). The responses to the questions utilized the same categories from Tables 3, 4, 5, and 6, yielding the following results subsequent to the implementation of the recommended analysis.

**Table 6.** Categorization of the outcomes for question 4.

Category	Explanation
Application in injury prevention and teaching	In this category, the focus is on physics as a means to help avoid mistakes and injuries, as well as being a booster of learning.
Management of forces in specific movements	In this category, an interaction of forces is mentioned, where an adjustment of forces in specific movements is required.

*Question 1.* How has your understanding of the physics of motion changed?

**Figure 6.**
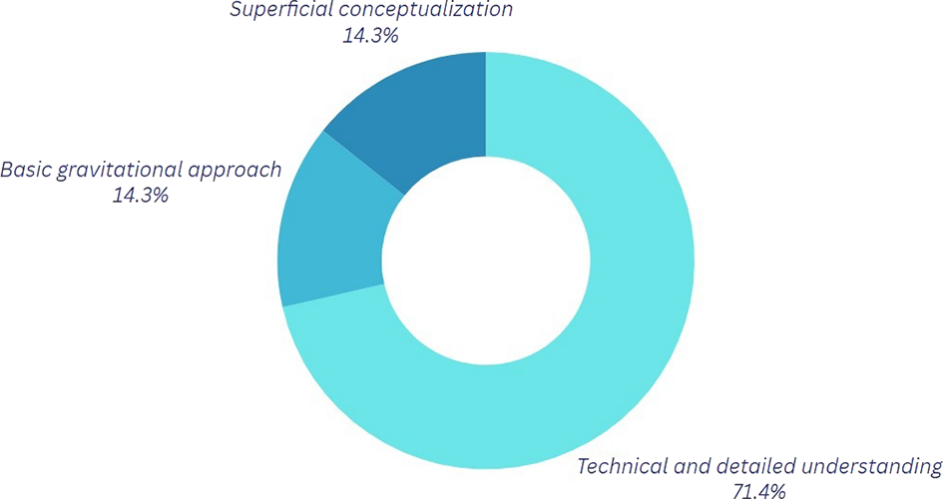
Graph corresponding to the answers to question 1, final phase.

Figure 6 illustrates a notable enhancement in the comprehension of the topic. Evidence indicates a conceptual appropriation of the concepts, with 71.4% of participants referencing elements such as forces, centripetal accelerations, center of mass, and force interactions, among others. The transition from basic or superficial conception to a more advanced categorization happens, demonstrating a distinct positive progression following the sessions and approach utilized, while also offering a more applicable perspective of physics. The favorable outcome is mostly attributable to the utilization of the Tracker program, which enabled participants to observe their motions from various perspectives, hence facilitating a more accurate analysis. A noteworthy point is made here; participants state that they typically videotape themselves from various perspectives to rectify problems, suggesting that this activity has been conducted previously. On this occasion, physical analysis was integrated through the program, enhancing the students’ analysis as they had become accustomed to viewing their motions from alternative angles.

*Question 2.* How have the analysis results altered your execution of specific movements?

**Figure 7.**
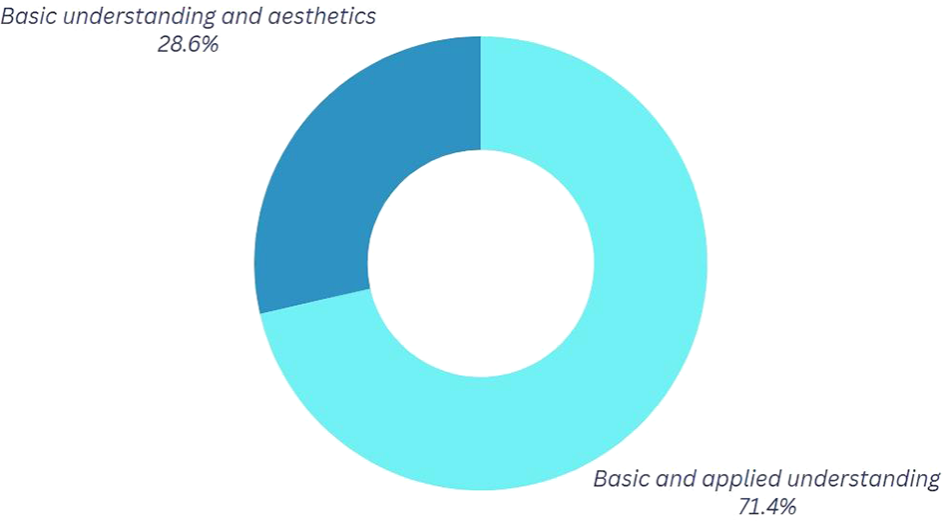
Graph corresponding to the answers to question 2, final phase.

Figure 7 reveals a notable shift towards the category “Technical and Applied Understanding.” 26.6% of participants maintain a connection between the mechanics of movement and aesthetics, whereas 71.4% indicate a technical and applied comprehension specifically pertaining to dance. As a result, following the sessions, participants can correlate physical characteristics to articulate their movements, while also acknowledging the application of physics in enhancing their performance.

*Question 3.* How are the analyzed physical variables related to your improvements in Break Dance?

**Figure 8.**
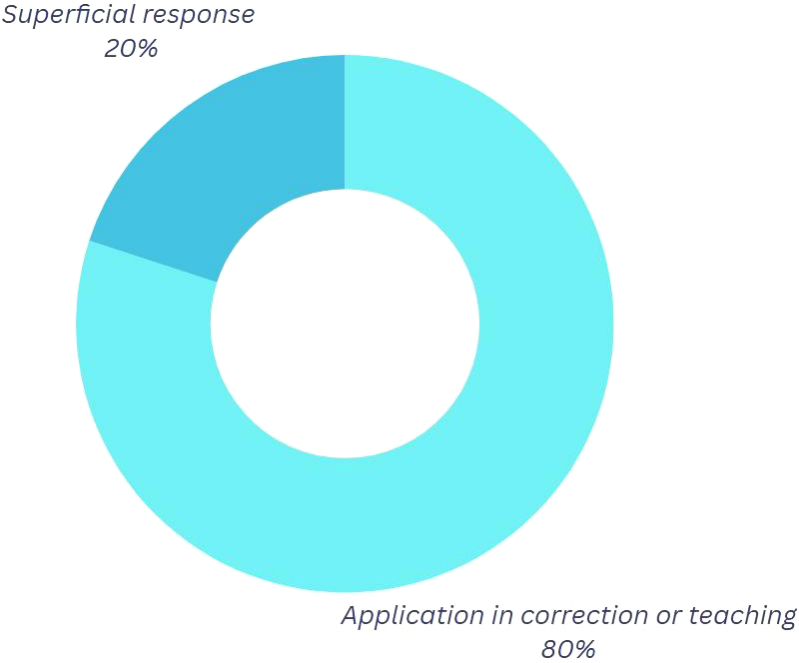
Graph corresponding to the answers to question 3, final phase.

In reference to Figure 8, the proportion of superficial replies diminished by fifty percent. The participants utilize the studied physical factors in their videos to rectify their dance routines. This indicates that they perceive physics as a beneficial component that can assist them in their practices and competitions, while also utilizing their knowledge to advise others, thereby fostering collaborative teaching within the team.

*Question 4.* How do you think this knowledge will influence your practice and execution of Break Dance?

**Figure 9.**
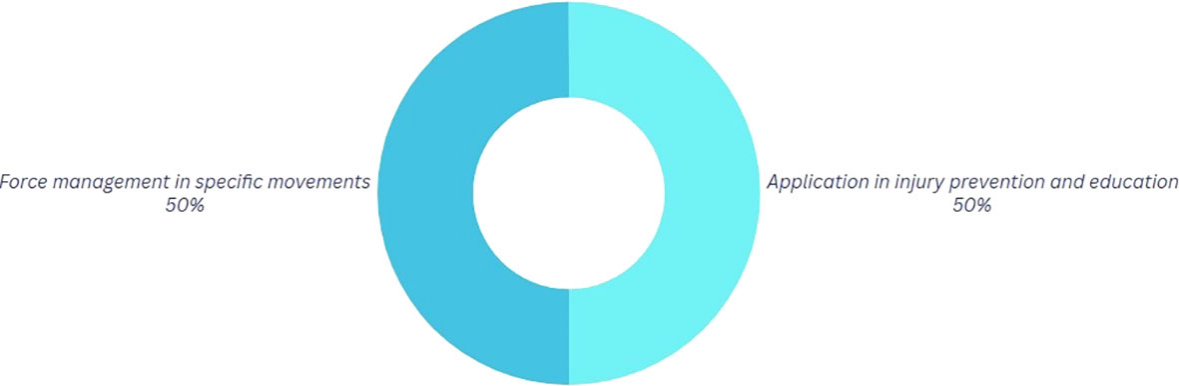
Graph corresponding to the answers to question 4, final phase.

Finally, the divergent opinions remain a factor evident in Figure 9. In comparison to phase 1, this percentage of participants remains unchanged, indicating that they perceive the relevance of knowledge when using it in their dancing practices. It can affect the prevention of errors in competitions, the execution of certain dance steps, and the attainment of enhanced proficiency. The implementation of this knowledge to prevent injuries and educate others is pertinent to 50% of the participants. This indicates that, despite varying characteristics, the gained knowledge remains beneficial to their profession.

On the other hand, the participants demonstrate that the implementation of the acquired knowledge is aimed at optimizing movement patterns. This finding suggests that computational physics constitutes a pedagogical tool of significant relevance in this specific context. Regarding the last variable analyzed, consistency was observed in both categories, indicating that the understanding of physics as a foundation for injury prevention and the improvement of both safety and movement quality remains constant regardless of the level of conceptual understanding. This suggests that the perception of safety remains stable without being conditioned by the degree of mastery of fundamental concepts.

## Discussion

The results indicate that Break Dance dancers have an innate tactile understanding of the physical principles underlying their movements, however they may first struggle to express it in scientific language. This conclusion aligns with the research by, ^8^ which suggests that physical engagement in dancing enhances the assimilation of tacit knowledge of physics, which can be articulated through suitable educational interventions. Consequently, embodied learning emerges as an efficacious method to connect practical experience with conceptual comprehension in unconventional educational settings.

Likewise, after the intervention with the Tracker software, most participants experienced a transition from a superficial understanding to a more technical and applied comprehension of physics in dance. This result aligns with the findings of, ^15^ who emphasize the importance of teachers encouraging reflection on the technical and kinesthetic aspects of movement, facilitating the crossing of “body thresholds” that allow for deeper learning. Moreover, it is recognized that the use of technology in these training processes allows it to be consolidated as an effective pedagogical tool for visualizing and analyzing abstract physical concepts through movement.

A relevant aspect identified in this study is the relationship between physical knowledge and injury prevention, as well as the improvement of artistic and technical performance. Previous research has shown that strength and conditioning training, based on scientific principles, not only prevents injuries but also enhances the aesthetic competence and artistic expression of dancers. ^16^ In this regard, the participants acknowledged that understanding the physics of their movements allows them to optimize their execution and reduce risks, which reinforces the practical usefulness of integrating science into dance training.

Finally, the results highlight the social and pedagogical value of sharing physical knowledge within the Break Dance community, promoting collaborative teaching and the cultural appropriation of scientific learning. According to, ^8, 17^ the amalgamation of science and art in physical education enhances conceptual comprehension and fortifies learners’ identity and motivation, fostering inclusive environments where knowledge is collaboratively constructed through bodily experience.

## Conclusions

The results of this research demonstrate that the integration of physics content in unconventional contexts, such as Breaking, through contextualized methodologies, constitutes a high-impact pedagogical strategy. The articulation between disciplinary knowledge and bodily practices not only allowed for a greater understanding of the physical phenomena associated with movement but also improved kinetic performance, injury prevention, and the enhancement of expressiveness in competitive settings. In this context, physics positions itself as an epistemological bridge that fosters a deeper understanding of biomechanics from a situated perspective.

Likewise, significant opportunities were identified for the design of specialized training programs that integrate scientific content with artistic practices, specifically dance. The variability observed in the levels of understanding among the participants highlights the need for differentiated pedagogical approaches, sensitive to previous educational trajectories. This diversity suggests that the meaningful appropriation of scientific knowledge can be enhanced through educational experiences that integrate art and movement, recognizing the body as a cognitive and expressive agent in the learning of science.

Finally, the integration of educational technology, such as the Tracker tool, emerged as a crucial pedagogical resource in facilitating the study of physics within creative and performative contexts. The visual examination of movement enhanced the comprehension of fundamental principles and encouraged a reframing of physics as a discipline that is pertinent, significant, and appropriate to artistic advancement. These findings advocate for the integration of interactive digital tools in transdisciplinary curricular initiatives, designed to foster a more inclusive, embodied, and contextualized scientific education.

## Ethics and consent

This research examined moral and legal rules concerning the ethical treatment and care of participants in the studies. ^9^ This study received approval from ACT 22-2022 (June 13, 2022) by the Research and Ethics Subcommittee of the Faculty of Education at the University of La Sabana. The document indicates that *“The Research and Ethics Subcommittee of the Faculty of Education has approved the project EDULCN-1-2022; Collaborative Learning, Computational Physics, and Experimental Setup Construction in the Approach to the Study of Renewable Energies, directed by Professor Diego Fernando Becerra (corresponding author). The suggestion is regarded favorably due to its significance. Under the guidance of project leader Professor Diego F. Becerra, his research focus within the Technologies for the Academy (Proventus) group, and his involvement in the Bachelor of Science in Natural Sciences and the Master’s program in Educational Innovation mediated by ICT”.* This study adheres to the global research conduct standards outlined in the UNESCO Universal Declaration on Bioethics and Human Rights, as well as the ethical principles set forth by the Ethics Committee of the University of La Sabana. The research emphasizes the importance of telling participants about the study, detailing their involvement, safeguarding their identity, and assuring that no bodily or emotional harm would ensue.

Moreover, it is stated that the research is undertaken for the advancement of the academic community, that the data is safeguarded and utilized exclusively for educational research purposes, and that a conscientious approach is maintained, remaining receptive to inquiries from participants and community members. Moreover, only data from minors whose parents have properly signed the informed permission form will be used into the study.

## Data Availability

No underlying data is associated with this article. Zenodo. Extended - Integration of Break Dance and Computational Physics. doi: https://doi.org/10.5281/zenodo.16782750. ^13^ The project contains the following extended data:
1. Systematization of data from the audio interview.docx 2. SISTEMATIZACION SEC ENGLISH.xlsx 3.
Figure 1.png 4.
Figure 2.jpeg 5.
Figure 3.jpeg 6.
Figure 4.jpeg 7.
Figure 5.jpeg 8.
Figure 6.jpeg 9.
Figure 7.jpeg 10.
Figure 8.jpeg 11.
Figure 9.jpeg 12.
Table 1.docx 13.
Table 2.docx Data are available under the terms of the Creative Commons Attribution 4.0 International license (CC-BY 4.0).
