# Supplementary material for: Integration of Breaking and Computational Physics: An Interdisciplinary Approach to Contextualized Teaching
Source: F1000Res. 2026 Feb 17;14:1129. Originally published 2025 Oct 17. [Version 2] doi: 10.12688/f1000research.166881.2 (PMC12780512; doi:10.12688/f1000research.166881.2)
Supplement: Peer review history

# Reviewer report: Reviewer response for version 2

**DOI:** 10.5256/f1000research.196155.r459296

**Published:** 2026-02-18

**Authors:** Claudia Steinberg, Sophie Manuela Lindner

**Affiliations:** 1 Institute fo dance and movement culture, German Sport University Cologne, Cologne, Germany; 2 Institute of dance and movement culture, German Sport University Cologne, Cologne, Germany

We have no comments to make.

Is the work clearly and accurately presented and does it cite the current literature?

Partly

If applicable, is the statistical analysis and its interpretation appropriate?

Yes

Are all the source data underlying the results available to ensure full reproducibility?

Yes

Is the study design appropriate and is the work technically sound?

Partly

Are the conclusions drawn adequately supported by the results?

Yes

Are sufficient details of methods and analysis provided to allow replication by others?

Partly

Reviewer Expertise:

Dance Scholar, Dance Science and Dance Pedagogy

We confirm that we have read this submission and believe that we have an appropriate level of expertise to confirm that it is of an acceptable scientific standard.

# Reviewer report: Reviewer response for version 1

**DOI:** 10.5256/f1000research.183933.r442782

**Published:** 2026-01-08

**Authors:** Yoav Bergner

**Affiliations:** 1 New York University, New York, New York, USA

This manuscript presents an innovative and culturally responsive approach to physics education that deserves recognition for its creative integration of breakdancing with computational analysis. The interdisciplinary framework is compelling, and the authors should be commended for working within a community-based setting with the "Soul Strong Crew" collective, demonstrating genuine commitment to contextualized and inclusive science education. The use of Tracker software to visualize biomechanical principles represents a thoughtful application of educational technology, and the qualitative findings suggesting shifts from intuitive to more technical understanding of physics concepts are promising.

 However, several areas require strengthening to fully realize the manuscript's potential. Most critically, the paper lacks an explicit theory of change—there is no clear articulation of the hypothesized mechanism by which video analysis of dance movements should lead to improved physics understanding, nor how constructivist or embodied learning principles are operationalized in this specific intervention. The pre- to post-intervention comparisons shown in pie charts (Figures 2-9) would be more effectively presented in tabular format, allowing readers to directly compare percentage shifts across categories and making the claimed improvements more transparent. Methodological details need expansion, particularly: (1) the specific procedures for developing and validating the thematic categories shown in Tables 3-6, including quotes and measures of inter-rater reliability; (2) detailed explanation of Tracker software operation, including which angles were recorded, how kinematic parameters were calculated, and how these quantitative data were integrated with qualitative responses.

Is the work clearly and accurately presented and does it cite the current literature?

Partly

If applicable, is the statistical analysis and its interpretation appropriate?

Not applicable

Are all the source data underlying the results available to ensure full reproducibility?

Yes

Is the study design appropriate and is the work technically sound?

Partly

Are the conclusions drawn adequately supported by the results?

Partly

Are sufficient details of methods and analysis provided to allow replication by others?

Yes

Reviewer Expertise:

Learning sciences, assessment, and computational methods in dance.

I confirm that I have read this submission and believe that I have an appropriate level of expertise to confirm that it is of an acceptable scientific standard, however I have significant reservations, as outlined above.

# Reviewer report: Reviewer response for version 1

**DOI:** 10.5256/f1000research.183933.r425750

**Published:** 2025-12-26

**Authors:** Jhonny Medina Paredes

**Affiliations:** 1 Universidad Austral de Chile, Valdivia, Chile

The research is well-structured and executed with scientific rigor; however, a more detailed qualitative analysis would be desirable to strengthen the conclusions. An example of this is the analysis of question 1 in phase 2. It states that there was a notable improvement in understanding the topic and that the evidence indicates a conceptual grasp of the concepts. I believe this evidence should be more explicit and detailed in the text.

 Finally, it should be noted that only 29% of the references used are from the last five years. This may be something that needs to be reviewed.

Is the work clearly and accurately presented and does it cite the current literature?

Yes

If applicable, is the statistical analysis and its interpretation appropriate?

Not applicable

Are all the source data underlying the results available to ensure full reproducibility?

Yes

Is the study design appropriate and is the work technically sound?

Yes

Are the conclusions drawn adequately supported by the results?

Yes

Are sufficient details of methods and analysis provided to allow replication by others?

Yes

Reviewer Expertise:

Physics Eduaction, STEM Education.

I confirm that I have read this submission and believe that I have an appropriate level of expertise to confirm that it is of an acceptable scientific standard.

# Reviewer report: Reviewer response for version 1

**DOI:** 10.5256/f1000research.183933.r428055

**Published:** 2025-12-23

**Authors:** Claudia Steinberg, Sophie Manuela Lindner

**Affiliations:** 1 Institute fo dance and movement culture, German Sport University Cologne, Cologne, Germany; 2 Institute of dance and movement culture, German Sport University Cologne, Cologne, Germany

Comments to: Is the work clearly and accurately presented, and does it cite the current literature?

 In the paper, both terms “Break Dance” and “Breaking” are used. Since “Break Dance” is the commercialized term, while “Breaking” is the official term (including in the context of the Olympic Games) and the term used within the Breaking community, we recommend using “Breaking” consistently throughout the paper, including in the title.

 p3: There is a disruption in the reading flow between the sentences “…technology computational methods that may enhance teaching and learning processes.” and “On the other hand, considering that the constructivist learning model seeks to encourage…”. It would be helpful to create a smoother transition to guide the reader more naturally into the study and maintain readability.

 Comments to: Is the study design appropriate and is the work technically sound?

 The research design is insufficiently described. In particular, the formation of categories does not appear to be adequately explained. Which mixed methods model was used (exploratory or explanatory mixed-method-design) (e.g. Creswell & Plano Clark 2018)? Which literature was referenced?

 Comments to: Are sufficient details of methods and analysis provided to allow replication by others?

 p3: Regarding the group of ten dancers, please specify how many were beginners and how many were experienced, since the participants’ experience ranges from 4 months to 9 years. This clarification is important for understanding the composition of the sample.

 P4: It would be beneficial to provide more detailed information about the Tracker video software. How exactly does the software work? Do videos need to be uploaded from different angles? How does the program perform measurement and evaluation? A clearer description of the tool and its functions would help readers understand the data collection and analysis process more thoroughly.

Is the work clearly and accurately presented and does it cite the current literature?

Partly

If applicable, is the statistical analysis and its interpretation appropriate?

Yes

Are all the source data underlying the results available to ensure full reproducibility?

Yes

Is the study design appropriate and is the work technically sound?

Partly

Are the conclusions drawn adequately supported by the results?

Yes

Are sufficient details of methods and analysis provided to allow replication by others?

Partly

Reviewer Expertise:

Dance Scholar, Dance Science and Dance Pedagogy

We confirm that we have read this submission and believe that we have an appropriate level of expertise to confirm that it is of an acceptable scientific standard, however we have significant reservations, as outlined above.

# Response

**Published:** 2026-02-03

**Authors:** Diego Fernando BECERRA RODRÍGUEZ

**Affiliations:** Education, Universidad de La Sabana, Chia, Cundinamarca, Colombia

Comments to: Is the work clearly and accurately presented, and does it cite the current literature?

**In the paper, both terms “Break Dance” and “Breaking” are used. Since “Break Dance” is the commercialized term, while “Breaking” is the official term (including in the context of the Olympic Games) and the term used within the Breaking community, we recommend using “Breaking” consistently throughout the paper, including in the title.**

 We appreciate your comments, which have significantly enhanced the text. The terms “Break Dance” and “Breaking” have been substituted in the title, introduction, research questions, study type, results analysis, and throughout the entire document. The phrase remained unchanged throughout the tools and interviews administered to the target demographic.

**p3: There is a disruption in the reading flow between the sentences “…technology computational methods that may enhance teaching and learning processes.” and “On the other hand, considering that the constructivist learning model seeks to encourage…”. It would be helpful to create a smoother transition to guide the reader more naturally into the study and maintain readability.**

 To enhance the document's readability, the last line of the paragraph, “Furthermore, these movements can be examined and assessed using computational methods that may enhance teaching and learning processes,” has been substituted. In this context, these movements can be analyzed and assessed by computational technologies, presenting the opportunity to enhance teaching methods in both dance and physics.

 Additionally, revisions were made to the transition from paragraph 3 to paragraph 4 of the introduction.

 Within a constructivist learning framework that prioritizes active student engagement and significant learning, particularly in physics education, the Tracker software facilitates the analysis of human body movement through its measurement and evaluation capabilities, rendering it an effective pedagogical tool for teaching physics.

**Comments to: Is the study design appropriate and is the work technically sound?**

**The research design is insufficiently described. In particular, the formation of categories does not appear to be adequately explained. Which mixed methods model was used (exploratory or explanatory mixed-method-design) (e.g. Creswell & Plano Clark 2018)? Which literature was referenced?**

 It is highlighted that the study adopts an exploratory approach, and the four main categories of analysis were included: (1) conceptual understanding of the physics of movement, (2) application of physics in movement execution and optimization, (3) pedagogical and formative dimension of physical knowledge, and (4) technology-mediated learning, safety, and transformation of participants’ perceptions. It is highlighted that the mixed-methods strategy facilitated the triangulation of physical, educational, and performative data, consistent with the framework for addressing complex educational issues thru methodological complementarity.

**Comments to: Are sufficient details of methods and analysis provided to allow replication by others?**

**p3: Regarding the group of ten dancers, please specify how many were beginners and how many were experienced, since the participants’ experience ranges from 4 months to 9 years. This clarification is important for understanding the composition of the sample.**

 At the end of the first paragraph of the methodology, the dancers’ tenure and experience have been included. Specifically, two dancers have been with the company for four months to one year, three possess one to three years of experience, two have three to six years, and three have been with the group for six to nine years.

**P4: It would be beneficial to provide more detailed information about the Tracker video software. How exactly does the software work? Do videos need to be uploaded from different angles? How does the program perform measurement and evaluation? A clearer description of the tool and its functions would help readers understand the data collection and analysis process more thoroughly.**

 The Tracker software is described as an open-source video analysis tool that is used to study motion through frame-by-frame analysis of recorded videos. The methodology includes a paragraph that outlines the request. Initially, the software facilitates the importation of standard video files and the calibration of spatial and temporal scales using reference points. This functionality enables the measurement of kinematic variables, such as position, displacement, velocity, acceleration, angular velocity, and center-of-mass trajectories. While it is not necessary to employ multiple camera angles, the utilization of various viewpoints can facilitate a more precise interpretation of intricate movements. Tracker then measures and evaluates selected body positions over time, resulting in the acquisition and visualization of variables such as trajectories, angular velocity, centripetal acceleration, and center of mass displacement through graphs. Tracker was employed in this investigation to facilitate data collection and analysis within an artistic and educational framework.
